# Expression of Abelson Interactor 1 (Abi1) Correlates with Inflammation, KRAS Mutation and Adenomatous Change during Colonic Carcinogenesis

**DOI:** 10.1371/journal.pone.0040671

**Published:** 2012-07-10

**Authors:** Konrad Steinestel, Silke Brüderlein, Julie Steinestel, Bruno Märkl, Michael J. Schwerer, Annette Arndt, Klaus Kraft, Christian Pröpper, Peter Möller

**Affiliations:** 1 Institute of Pathology, University of Ulm, Ulm, Germany; 2 Department of Pathology, Bundeswehrkrankenhaus Ulm, Ulm, Germany; 3 Department of Urology, University of Ulm, Ulm, Germany; 4 Institute of Pathology, Klinikum Augsburg, Augsburg, Germany; 5 Department of Forensic Medicine/Aircraft Accident Investigation, German Air Force Institute of Aviation Medicine, Fürstenfeldbruck, Germany; 6 Institute of Anatomy and Cell Biology, University of Ulm, Ulm, Germany; Vanderbilt University Medical Center, United States of America

## Abstract

**Background:**

Abelson interactor 1 (Abi1) is an important regulator of actin dynamics during cytoskeletal reorganization. In this study, our aim was to investigate the expression of Abi1 in colonic mucosa with and without inflammation, colonic polyps, colorectal carcinomas (CRC) and metastases as well as in CRC cell lines with respect to BRAF/KRAS mutation status and to find out whether introduction of KRAS mutation or stimulation with TNFalpha enhances Abi1 protein expression in CRC cells.

**Methodology/Principal Findings:**

We immunohistochemically analyzed Abi1 protein expression in 126 tissue specimens from 95 patients and in 5 colorectal carcinoma cell lines with different mutation status by western immunoblotting. We found that Abi1 expression correlated positively with KRAS, but not BRAF mutation status in the examined tissue samples. Furthermore, Abi1 is overexpressed in inflammatory mucosa, sessile serrated polyps and adenomas, tubular adenomas, invasive CRC and CRC metastasis when compared to healthy mucosa and BRAF-mutated as well as KRAS wild-type hyperplastic polyps. Abi1 expression in carcinoma was independent of microsatellite stability of the tumor. Abi1 protein expression correlated with KRAS mutation in the analyzed CRC cell lines, and upregulation of Abi1 could be induced by TNFalpha treatment as well as transfection of wild-type CRC cells with mutant KRAS. The overexpression of Abi1 could be abolished by treatment with the PI3K-inhibitor Wortmannin after KRAS transfection.

**Conclusions/Significance:**

Our results support a role for Abi1 as a downstream target of inflammatory response and adenomatous change as well as oncogenic KRAS mutation via PI3K, but not BRAF activation. Furthermore, they highlight a possible role for Abi1 as a marker for early KRAS mutation in hyperplastic polyps. Since the protein is a key player in actin dynamics, our data encourages further studies concerning the exact role of Abi1 in actin reorganization upon enhanced KRAS/PI3K signalling during colonic tumorigenesis.

## Introduction

Worldwide, death from intestinal neoplasms is a high ranking cause of death from cancer in both male and female populations. Up to date, fundamental insights into the molecular biology of tumorigenesis, growth and metastasis were obtained, leading to today’s sight of an interplay of environmental and genetic causes finally resulting in the disease along a multi-step process [1], [2], [3]. In recent years, different pathways of colorectal carcinogenesis have been proposed and thoroughly discussed. It has become clear that certain molecular features, such as APC-, KRAS-, or BRAF-mutations or microsatellite instability are associated with different pathways of tumorigenesis, leading to clinically and morphologically distinct colorectal carcinoma entities [4], [5]. The clinical significance of these molecular alterations and their exact role in tumorigenesis are still subject to ongoing research [6]. However, it has been shown that reorganization of the actin cytoskeleton is an important step in colorectal tumorigenesis, and that the expression patterns of proteins associated with this reorganization are significantly altered during the progression from colorectal adenoma to carcinoma [7]. Abi1, a 65 kD substrate of the Abelson tyrosine kinase and component of a trimeric complex consisting of Abi1 and its protein interaction partners Eps8 and Sos1, is a known adaptor protein involved in actin reorganization and lamellopodia formation. It has been shown to mediate cell spreading and migration by interacting with WASP-family verprolin-homologous protein 2 (WAVE2) [8], [9]. Interestingly, Abi1 has also been shown to act in a multiprotein complex together with the EGFR/RAS-signalling effector molecule Phosphatidylinositol-3-kinase (PI3K). The Abi1/PI3K/Eps8/Sos1 complex facilitates actin reorganization via activation of Rac [10]. To mediate its function, Abi1 localizes at the cellular leading edge of fibroblasts. In neurons, it is located at the postsynaptic density of maturating synapses, where it supports synaptic maturation and establishment of the postsynaptic density (PSD) in early neurogenesis [11], [12], [13]. In these studies, it has further been shown that Abi1 translocates from the synapse to the nucleus after neuronal stimulation and can be precipitated from the nucleus in a complex with the Myc/Max transcription factor. This highlights the possibility of an additional, nuclear role of Abi1 in transcriptional regulation as a part of this transcription-factor complex.

In tumor tissue and cell lines, it has been shown that Abi1 contributes to leukemogenic potential in leukemic cells expressing oncogenic Bcr/Abl and v-Abl. RNAi knockdown of Abi1 in these cells led to impaired cell migration and abnormal actin remodeling and it has been suggested that these effects might be mediated through Src family kinases [14]. Consistent with this data, Abi1 seems to be overexpressed in highly invasive breast cancer cell lines compared to weakly invasive ones, and Abi1 knockdown in these cells also led to decreased invasiveness and migration ability [15]. Interestingly, our work group recently identified the heterogeneous nuclear ribonucleoprotein K (hnRNP K) as one protein interaction partner of Abi1, while overexpression and aberrant localization of hnRNP K in tumor cells has previously been described to be correlated with adverse outcome in colorectal carcinoma patients [16], [17].

In this study, we analyzed the expression pattern of Abi1 in colonic mucosa with and without inflammation, in colonic precursor lesions, colorectal carcinoma and colorectal cancer metastasis and correlated Abi1 expression with the respective KRAS/BRAF mutation status of each lesion. Furthermore, we analyzed Abi1 expression status in three widely-used colorectal carcinoma cell lines with known mutation status as well as in two colorectal carcinoma cell lines that have been previously established by our work group [18], [19]. Finally, we investigated the effect of transfection with either KRAS wild-type or KRAS G12D as well as TNFalpha stimulation and Wortmannin treatment on Abi1 expression in KRAS wild-type colorectal carcinoma cells.

## Results

### Clinicopathological Data

In general, patients with hyperplastic polyps (HPP) and sessile serrated polyps/adenomas (SSA/P) were younger (60±17.5 y and 59±15.3 y, Table 1) and more SSA/P were localized in the right colon (R:L 12∶8). Patients with traditional serrated adenomas and tubular adenomas were older (78±9.8 y and 64±5.5 y) and the lesions were localized in the left rather than in the right colon (R:L 2∶6 and 4∶9). In general, SSA/P tended to be more expanded (12 of 20 larger than 5 mm).

**Table 1:** Clinico-pathologic characteristics of analyzed samples.

			HealthyMucosa	Inflamed Mucosa	HPP	SSP/A	TSA	TbA	Ca	Met
Total Patients (n = 95)			23	4	12	14	5	8	20	9
Total samples (n = 126)			24	9	23	20	8	13	20	9
Age			69.5±17.7	23±21.9	60±17.5	59±15.3	78±9.8	64±5.5	68.5±10,1	60±7,7
Gender (M:F)			13∶10	3∶1	7∶5	5∶9	1∶4	5∶3	12∶8	4∶2
Location (R:L)			13∶11	3∶6	11∶12	13∶7	2∶6	4∶9	6∶14	–
Size (mm)		<5	–	–	11	8	2	1	–	–
		≥5	–	–	12	12	6	12	–	–
BRAF (c600)			n.t.	n.t.	6	14	.0	1	1	0
KRAS (c12/13)			n.t.	n.t.	8	3	4	4	10	2
MSS			n.t.	n.t.	23/23	20/20	8/8	13/13	17/20	9/9
Grading	Well		–	–	–	–	–	–	4	
	Moderate		–	–	–	–	–	–	10	6
	Poor		–	–	–	–	–	–	6	3
Stage	Dukes A		–	–	–	–	–	–	3	–
	Dukes B		–	–	–	–	–	–	6	–
	Dukes C		–	–	–	–	–	–	9	–
	Dukes D unknown								0	9
									2	
Site	Liver		–	–	–	–	–	–	–	6
	Lung		–	–	–	–	–	–	–	2
	Others		–	–	–	–	–	–	–	1

Abbreviations: HPP: hyperplastic polyp; SSA/P: sessile serrated polyp/adenoma; TSA: traditional serrated adenoma; TbA: tubular adenoma; Ca: invasive colorectal carcinoma; Met: Metastasis; BRAF c600: B1 Rapidly accelerated fibrosarcoma codon 600 mutation; KRAS c12/13: Kirsten rat sarcoma codon 12 or 13 mutation.; n.t.: not tested; -: not applicable.

### KRAS and BRAF Mutation Status of Precursor Lesions and Colorectal Carcinomas

KRAS codon 12 mutations were found in 8/23 hyperplastic polyps (34.8%) and 3/20 sessile serrated polyps/adenomas (15%, Table 1; exact mutations are shown in Table S1). BRAF V600E mutation was detected in 6/23 hyperplastic polyps (26%) and 14/20 sessile serrated polyps/adenomas (70%). We found KRAS codon 12 and 13 mutations in half of the traditional serrated adenomas (4/8, 50%) but could not detect a BRAF mutation in these lesions. 4 of 13 tubular adenomas (30.8%) carried a KRAS codon 12 or 13 mutation, while only one tubular adenoma carried a BRAF codon 600 mutation (7.7%). Among the colorectal carcinoma specimens, we detected a KRAS codon 12 or 13 mutation in half of the samples (10/20, 50%), but a BRAF codon 600 mutation only in one sample (1/20, 5%). Two of the KRAS-mutated and the single BRAF-mutated carcinoma showed loss of nuclear MLH-1 and PMS-2 immunostaining. Two of nine metastases (25%) carried a KRAS codon 12 mutation. KRAS and BRAF mutations were mutually exclusive. There was no correlation between mutation status and either size or origin of the specimen (p>0.1).

### Abi1 Expression in Healthy and Inflamed Mucosa

**Figure 1:**
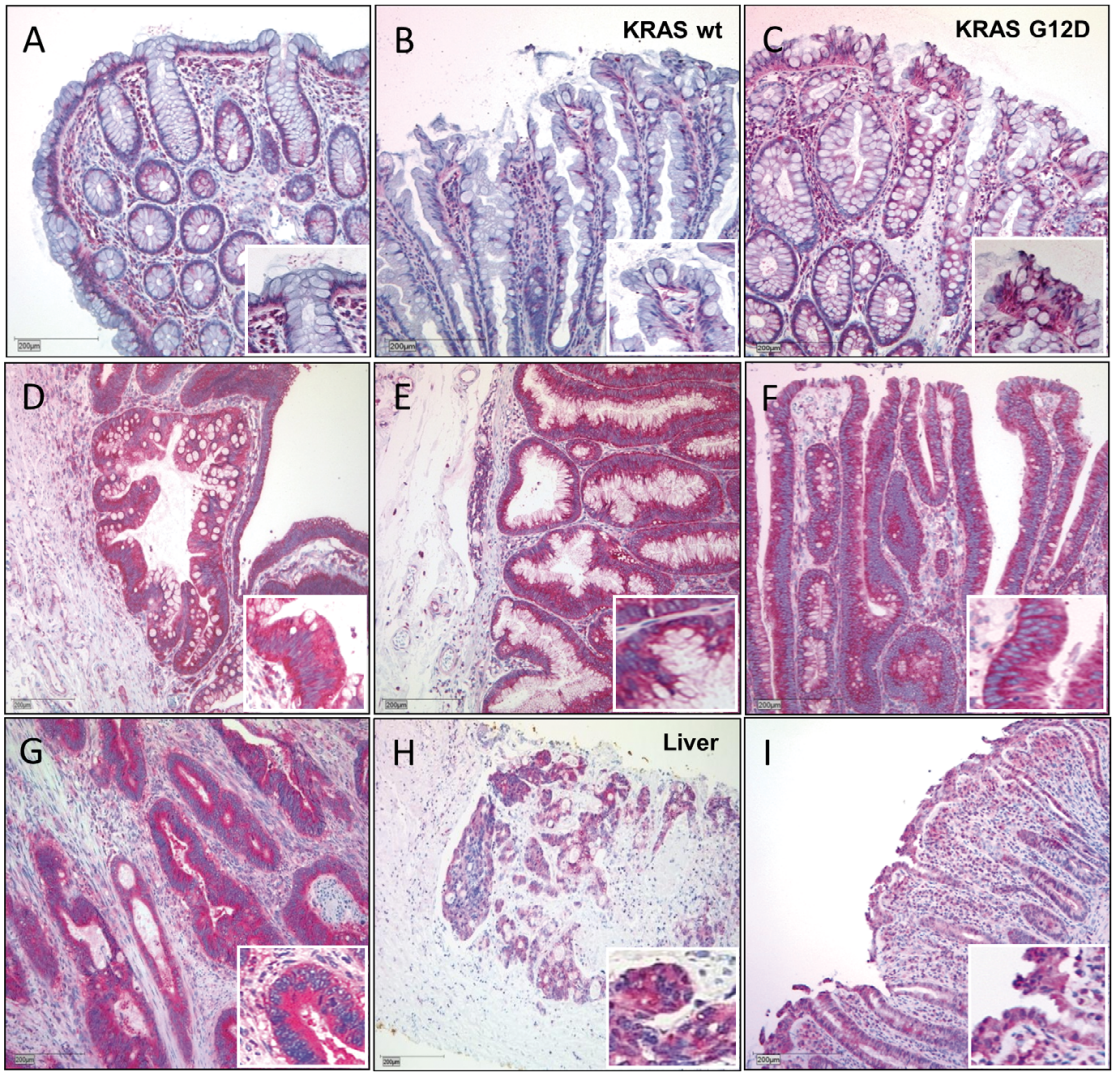
Abi1 immunohistochemistry in colorectal tissue samples. A and **B**, Regular mucosa (A) and wild-type hyperplastic polyps (B) show only moderate, cytoplasmatic and basally located Abi1 immunoreactivity. Note intense staining of lymphocytes in subjacent stroma. **C**, Overexpression of Abi1 in a representative hyperplastic polyp harboring KRAS G12D mutation. **D-F**, Intense immunoreactivity in (wild-type) sessile serrated adenoma (D), traditional serrated adenoma (E) and tubular adenoma (F). Note that positivity for Abi1 is exclusively cytoplasmic. **G** and **H,** strong immunoreactivity for Abi1 in invasive colorectal carcinoma and in a liver metastasis of colorectal carcinoma. **I,** strong cytoplasmic immunoreactivity for Abi1 in inflamed colonic mucosa. *Stain: anti-Abi1, haematoxylin; Bar indicates 200* *µm*.

**Figure 2:**
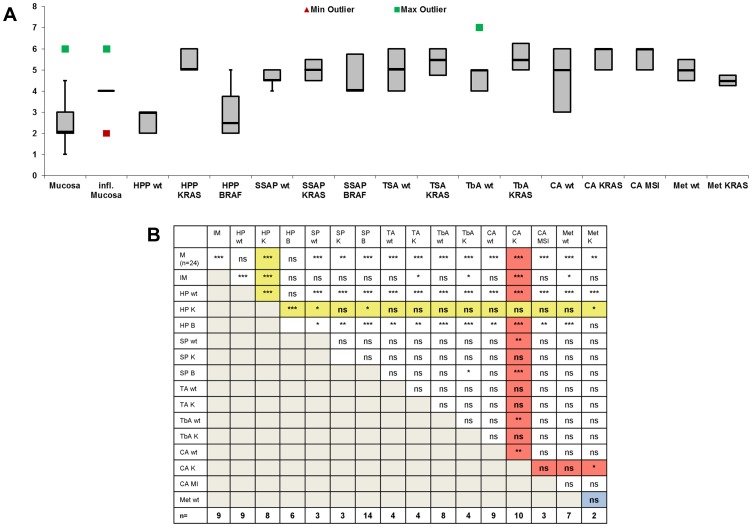
Abi1 expression analysis in specimens and cell lysates. A , Distribution of Abi1 expression in healthy and inflamed mucosa, hyperplastic polyps (HPP), sessile serrated polyps/adenomas (SSA/P), traditional serrated adenomas (TSA), tubular adenomas (TbA), invasive colorectal carcinoma (Ca) and metastases (Met). All values except BRAF-mutated TbA and carcinoma (each n = 1) are shown in box and whisker plot. Green squares represent maximum outliers, red squares represent minimum outliers. For inflamed mucosa, median, 1^st^ and 3^rd^ quartile are equal (score = 4). **B**, Statistical differences in Abi1 expression among all examined tissue specimens with respect to mutation status and, where applicable, microsatellite stability of each lesion. The lane for KRAS-mutated HPP is highlighted with a yellow background, the lane for KRAS-mutated invasive carcinoma is highlighted with a red background. The undermost line shows the number of examined samples in each group. M: healthy mucosa; IM: inflamed mucosa; HP wt, HP K, HP B: wild-type, KRAS-mutated and BRAF-mutated hyperplastic polyps; SP wt, SP K, SP B: wild-type, KRAS-mutated and BRAF-mutated sessile serrated polyps/adenomas; TA wt, TA K: wild-type and KRAS-mutated traditional serrated adenomas; TbA wt, TbA K: wild-type and KRAS-mutated tubular adenomas; CA wt, CA K, CA MI: wild-type, KRAS-mutated and microsatellite-instable carcinomas; Met wt, Met K: wild-type and KRAS-mutated metastases; n.s.: not significant; *p<0.1;** p<0.05;*** p<0.01.

Abi1 expression score was 2.46±1.15 in healthy mucosa (Muc) and significantly higher (4.0±1.05) in inflamed mucosa (IM; Table 2, Fig. 1A, I and Fig. 2; p<0.01, all exact *p*-values are shown in Fig. S4). Abi1 staining was localized in the basal cytoplasm in healthy mucosa, while inflamed mucosa showed a heterogeneous staining pattern of almost no positivity to strong, ubiquitous cytoplasmic positivity (Fig. 1 I). Inflammatory cells in underlying stroma and lymphoid follicles were strongly positive. There was no statistical significant difference between specimens from the right (3.19±1.52) or left colon (2.59±1.12; p>0.1).

**Table 2:** Abi1 expression in analyzed samples.

		Abi1 score	± SD	n
Healthy Mucosa (HM)		2.46	1.15	24
Right Colon (R)		3.19	1.52	13
Left colon (L)		2.59	1.12	11
Inflamed Mucosa (IM)		4	1.05	9
HPP				
	HPP wt	2.66	0.5	9
	HPP BRAFc600	3	1.26	6
	HPP KRAS c12/13	5.38	0.52	8
SSA/P	SSA/P wt	4.66	0.58	3
	SSA/P BRAF c600	4.64	1.01	14
	SSA/P KRAS c12/13	5	1	3
TSA	TSA wt	5	1.15	4
	TSA KRAS c12/13	5.25	0.96	4
TbA	TbA wt	4.86	0.99	8
	TbA BRAF c600	5	n.a.	1
	TbA KRAS c12/13	5.75	0.96	4
Ca	Ca wt	4.56	1.33	9
	Ca BRAF c600	3	n.a.	1
	Ca KRAS c12/13	5.8	0.79	10
	Ca MSI	5.33	1.15	3
Met	Met wt	5.14	1.07	7
	Met KRAS c12/13	4.5	0.7	2

All values shown as mean ± SD; abbreviations: HPP: hyperplastic polyp; SSA/P: sessile serrated polyp/adenoma; TSA: traditional serrated adenoma; TbA: tubular adenoma; Ca: invasive colorectal carcinoma; Met: Metastasis; BRAF c600: B1 Rapidly accelerated fibrosarcoma codon 600 mutation; KRAS c12/13: Kirsten rat sarcoma codon 12/13 mutationMSI: microsatellite instable tumors; n: number of examined samples; n.a.: not applicable due to low sample number.

### Abi1 Expression in Serrated Lesions

**Figure 3:**
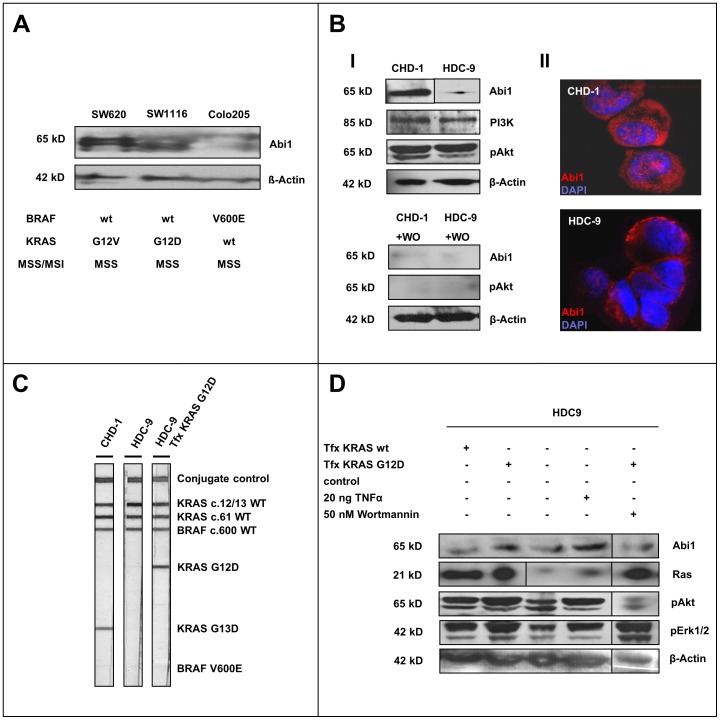
Abi1 in colorectal cancer cell lines. A , Abi1 immunoblotting of colorectal carcinoma whole cell line lysates with different KRAS/BRAF mutation status shows upregulation of Abi1 in KRAS-mutated SW620 and SW1116, but only a faint signal in BRAF-mutated Colo205 cells. **B**, Immunoblotting of CHD-1 and HDC-9 cell lysates show overexpression of Abi1 in CHD1 cells, while both cell lines express comparable amounts of PI3K (I). There is slightly stronger Akt phosphorylation in CHD1 compared to HDC9 cells. Application of 50 nM Wortmannin (WO) almost completely repressed the pAkt and Abi1 signals. Immunofluorescence microscopy shows strong cytoplasmatic and nuclear Abi1 staining in CHD-1 cells, but only a faint cytoplasmatic signal in HDC-9 cells (II). **C,** KRAS/BRAF mutation testing reveals an activating KRAS G13D mutation in CHD-1 (left lane), while HDC-9 cells are KRAS wild-type (central lane). Transfection of HDC-9 cells with a KRAS G12D-construct leads to appearance of a band indicating a KRAS G12D-mutation (right lane). Both cell lines are BRAF wild-type. **D,** Immunoblotting of HDC-9 after transfection, TNFalpha and Wortmannin treatment shows an increase in pErk1/2 and pAkt and overexpression of Abi1 upon transfection with constitutively active KRAS G12D (2^nd^ lane) compared to both control (3^rd^ lane) and to transfection with wild-type KRAS (1^st^ lane). Stimulation with TNFalpha also enhances phosphorylation of signaling proteins and leads to upregulation of Abi1 (4^th^ lane). The overexpression of Abi1 could be reversed by application of 50 nM Wortmannin (5^th^ lane).

Abi1 expression patterns in HPP varied from no expression to strong, ubiquitous cytoplasmic positivity. Again, underlying lymphocytes showed strong Abi1 immunoreactivity. Considering mutation status, there was no significant Abi1 overexpression in HPP without one of the tested mutations (Table 2, 2.66±0.5) compared to healthy mucosa (p>0.1), but there was significant lower Abi1 expression score in wild-type HPP compared to inflamed mucosa (Table 2, Fig. 1 B,I, Fig. 2, p<0.01). HPP with BRAF V600E mutation (3±1.26) were not significantly different from wild-type polyps (p>0.1). In polyps harboring KRAS mutations, however, mucosal cytoplasmic Abi1 expression score was significantly increased compared to healthy mucosa and to wild-type as well as BRAF-mutated HPP (Fig. 3 C, 5.38±0.52 vs. 2.46±1.15 or 2.66±0.47 or 3±1.26 (p<0.01); Fig. 1 C, Fig. 2– yellow background). Compared to inflamed mucosa, there was a statistically significant overexpression of Abi1 (Table 2, Fig. 1 I, Fig. 2, and Fig. S4, p<0.01). To examine whether this overexpression in KRAS-mutated HPP was due to an increase in proliferative activity, we also performed Ki67 immunostaining, but did not find an enhancement of the proliferative zone in KRAS-mutated polyps (Fig. S1). There was no statistically significant difference between Abi1 expression in smaller (<0.5 cm; 3.45±1.29) and larger (≥0.5 cm; 3.91±1.62) HPP (p>0.1).

In wild-type sessile serrated polyps/adenomas, Abi1 expression was 4.66±0.58, and therefore significantly higher compared to healthy mucosa (p<0.01; Fig. 1 A, D and Fig. 2) but not to inflamed mucosa (Table 2, p>0.1; Fig. 1 D and I). Again, there was no statistically significant difference between Abi1 expression in smaller (<0.5 cm; 4.85±1.57) and larger (≥0.5 cm; 4.23±1.42) SSA/P (p>0.1). Abi1 showed consistent and strong immunoreactivity in mucosal cytoplasm and underlying lymphocytes. Considering BRAF mutation status, Abi1 expression in SSP/A was not significant higher in BRAF-mutated lesions (4.64±1.01) compared to wild-type or KRAS-mutated lesions (Table 2, Fig. 2, p>0.1). Abi1 immunoreactivity was higher in all SSA/P compared to wild-type and BRAF-mutated HPP, although only slightly significant (p<0.1). Compared to KRAS-mutated HPP, there was no difference in Abi1 expression (p>0.1, Fig. 2 B). Wild-type traditional serrated adenomas had a significantly higher mucosal Abi1 expression score compared to healthy mucosa, wild-type and BRAF-mutated HPP, but not to inflamed mucosa and to KRAS-mutated HPP (Table 2, 5.0±1.15; p<0.01 and p>0.1, respectively; Fig. 1E and I, Fig. 2). Among TSAs, there was no difference between KRAS-mutated and wild-type lesions, but KRAS-mutated TSA had a slightly higher Abi1 expression compared to IM (5.25±0.96; p<0.1). There was no significant difference in Abi1 expression between SSA/P and TSA in general, and there was no significant correlation between Abi1 expression and either size (4±2.82 vs. 5.16±0.98 while only two TSA were smaller than 0.5 cm) or origin of the specimen (p>0.1).

### Abi1 Expression in Tubular Adenomas (TbA)

Abi1 was significantly overexpressed in KRAS-mutated TbA compared to both healthy mucosa or inflamed mucosa (Table 2, 5.75±0.96, p<0.01 and p<0.1; Fig. 1 F and I, Fig. 2). There was also a significant Abi1-overexpression in wild-type TbA (4.86±0.99) compared to healthy mucosa, but not to inflamed mucosa (p>0.1). There was no statistically significant difference between Abi1 expression in smaller (<0.5 cm; 5.66±1.15) and larger (≥0.5 cm; 5±0.94) TbA (p>0.1). Compared to HPP, Abi1 expression was stronger in all TbA compared to wild-type and BRAF-mutated HPP, but similar to KRAS-mutated HPP (p<0.01 and p>0.1, respectively; Fig. 1B–F, Fig. 2). Again, Abi1 showed strong immunoreactivity in mucosal cytoplasm and underlying lymphocytes. While there was a slight, but significant overexpression of Abi1 in TbA compared to BRAF-mutated SSA/P, there was no difference between TbA and other SSA/P or TSA (p<0.1 and p>0.1, respectively). We only had one BRAF-mutated TbA in the study, and could therefore not perform statistical testing with this entity.

### Abi1 Expression in Colorectal Carcinoma and Metastasis

Abi1 was strongly and ubiquitously expressed in the cytoplasm of most examined tumor samples and metastases. Again, there was a slight, but statistical significant overexpression of Abi1 in the KRAS-mutated tumors compared to the wild-type group (Table 2, 5.8±0.79 vs. 4.56±1.33, p<0.05, Fig. 1 G,H and Fig. 2). We only had one BRAF-mutated tumor in the study, and could therefore not perform statistical testing with this entity. Interestingly, Abi1 expression in KRAS-mutated carcinomas was also higher compared to mucosa, inflamed mucosa, wild-type HPP, SSA/P and TbA as well as BRAF-mutated SSA/P, but not different from all TSAs and KRAS-mutated HPP, SSA/P and TbA (Fig. 2, orange background; see Fig. S4 for exact *p*-values). There was no significant difference between specimens from the right (5.0±1.27) or left colon (5.2±1.25, p>0.1) or related to tumor grade (well and moderate: 5.09±1.32; poor: 4.96±1.08, respectively; p>0.1). Furthermore, there was no significant difference in Abi1 expression between microsatellite stable (MSS, n = 17) and instable (MSI, n = 3) tumors (p>0.1). The expression scores in wild-type carcinoma (regardless of microsatellite stability) and metastases were significantly higher compared to healthy mucosa, wild-type and BRAF-mutated HPP (p<0.05). The difference in Abi1 expression between KRAS-muteted carcinoma and KRAS-mutated HPP, on the other hand, was not significant (Fig. 2, p>0.1). Furthermore, the score was not significantly different in carcinomas and metastases than in TSA (p>0.1). Among colorectal carcinoma metastases, Abi1 immunohistochemistry showed the same intense cytoplasmic staining pattern in almost all examined samples (Fig. 1H). However, there was a statistically significant decrease in Abi1 expression in KRAS-mutated metastases compared to KRAS-mutated primary tumors and HPP (Table 2, 4.5±0.7; Fig. 2, p<0.1) There was no significant difference in Abi1 expression score related to the site of metastasis (p>0.1).

### Abi1 Expression in Colorectal Carcinoma Cell Lines SW620, SW1116 and Colo205

Western blotting showed only very faint bands detected at 65 kD in BRAF-V600E-mutated Colo205 cells, but a strong double-band in KRAS G12V-mutated SW620 and KRAS G12D-mutated SW1116 cells, respectively (Fig. 3A, upper panel). Equal protein loading was shown by ß-Actin immunoblotting (lower panel). The blot therefore shows a higher expression of Abi1 in the cell lines harboring KRAS G12V and G12D mutations, but not in the cell line harboring the BRAF V600E mutation.

### Abi1 Expression and KRAS/BRAF Mutation Testing in CHD-1 and HDC-9 Colorectal Carcinoma Cells

Western blotting with an antibody against Abi1 showed a strong band at 65 kD in CHD-1 cells, but only a weak signal in HDC-9 cells (Fig. 3B, I, upper panel). Equal amounts of protein were detected with antibodies against PI3K (85 kD) and ß-Actin (42 kD) in both cell lysates, while CHD-1 cells showed a slightly stronger signal for phosphorylated Akt. Treatment of both cell lines with 50 nM of the PI3K-inhibitor Wortmannin for 72 hours resulted in an absence of phosphorylated Akt compared to untreated cells and an almost complete extinction of the Abi1 signal in both cell lines (Lower panels). Immunofluorescence microscopy showed strong cytoplasmatic and nuclear Abi1 expression in CHD-1 cells (Fig. 3B, II, upper image) compared to a weak cytoplasmatic signal in HDC-9 cells (lower image). Strip-assay-based KRAS/BRAF mutation analysis revealed an activating G13D mutation in the CHD-1 cell line (Fig. 3C, left lane) that could be tracked to a GGC to GAC point mutation in codon 13 by subsequent pyrosequencing (Fig. S3, top left pyrogram). The HDC-9 cell line turned out to carry wild-type KRAS (Fig. 3C, central lane and Fig. S3, central left pyrogram). Both cell lines are BRAF codon 600 wild-type as confirmed by strip assay testing and pyrosequencing (Fig. 3C and Fig S3, right pyrograms).

### Transfection Experiments and TNFalpha Treatment in CHD-1 and HDC-9 Colorectal Carcinoma Cells

We transfected pcDNA3.1 mammalian expression vectors carrying either wild-type KRAS or G12D-mutated KRAS into KRAS wild-type HDC-9 cells. Overexpression of KRAS was shown by pan-Ras immunoblotting (Fig. 3D), while introduction of the G12D mutation was confirmed by KRAS/BRAF strip assay testing and pyrosequencing (Fig. 3C, right lane and Fig. S3, lower left pyrogram). Transfection with the KRAS G12D construct led to an upregulation of Abi1 as confirmed by western immunoblotting, while there was no increase in protein expression upon transfection with the wild-type KRAS construct (Fig. 3D, upper panel, left lanes). Transfection with wild-type KRAS led to an increase in Akt and Erk1/2 phosphorylation compared to the control lysate (central panels). The pAkt and pErk1/2 signals were stronger after KRAS G12D transfection. We then treated HDC-9 cells with 20 ng TNFalpha to simulate an inflammatory setting. This led to a strong upregulation of Abi1 as was again confirmed by western blotting (Fig. 3D, upper panel, right). TNFalpha treatment also enhanced phosphorylation of Akt and Erk1/2. To show whether Abi1-upregulation depends on PI3K activity, we treated HDC-9 cells that had previously been transfected with KRAS G12D with 50 nM of the PI3K-inhibitor Wortmannin for 72 hours. This led to a strong reduction in Abi1-expression compared to the transfected cells (Fig. 3D, upper panel, far right).

Taken together, these results show an increase in MAPK/PI3K signaling and an overexpression of Abi1 upon transfection of wild-type HDC-9 colonic carcinoma cells with constitutively active KRAS G12D. This upregulation can be hindered by application of the PI3K-inhibitor Wortmannin. Furthermore, stimulation with TNFalpha enhances phosphorylation of signaling proteins and also leads to upregulation of Abi1.

## Discussion

### Semiquantitative Abi1 Expression Analysis

In this study, we firstly analyzed the expression and distribution of Abi1, a protein described as an important regulator of actin dynamics, in healthy and inflamed colonic mucosa and precursor lesions as well as colorectal adenocarcinoma and metastasis. Of course, it cannot be guaranteed that our criteria of inclusion and exclusion of samples (as described in the Materials and Methods section) did not cause selection bias among the different diagnosis groups, and some of the groups (eg. TSA and metastases) are too small for epidemiologic evaluation. However, it was not the aim of this study to carry out an extensive epidemiologic analysis of colonic precursor lesions- all the more since there have been published excellent studies on this topic [20], [21]- but to analyze Abi1 expression in many different colonic lesions. Clinico-pathologic analysis of our sample collection showed that patients with serrated colonic lesions tended to be younger and that the lesions were localized in the right rather than in the left colon. These findings are consistent with previously obtained data from the literature and, although we did not include a vast number of samples, they support the assumption that the analyzed collection is representative [4]. The frequency of both BRAF and KRAS mutations in different colorectal precursor lesions in our study group reflects data from the literature [22], [23], [24], [25], [26], [27].

Immunohistochemically, we found weak and basal Abi1 expression in healthy colonic epithelium, with no staining signal in the nucleus. This localization pattern might be due to an interaction with basally localized integrins, since interaction of Abi1 with both alpha4 and beta1 integrin has been previously described [28], [29]. The strong Abi1 staining of underlying, interstitial inflammatory cells has also been previously described and is a helpful positive control [30]. In colonic biopsies with inflammation, there was a significantly stronger staining signal. With regard to BRAF and KRAS mutation status in colonic precursor lesions and invasive carcinomas, wefound that KRAS-mutated HPP showed significantly higher Abi1 expression compared to healthy and inflamed mucosa as well as wild-type and BRAF-mutated HPP. This is interesting because it has been previously proposed that some sorts of HPP might in fact represent precursor lesions during the serrated pathway of colon carcinogensis [22]. The upregulation of Abi1 in KRAS-mutated but not BRAF-mutated HPP was not due to an increase in proliferative activity, since Ki67 staining showed no enhancement of the basal proliferative zone in KRAS-mutated HPP compared to wild-type HPP. This finding is consistent with a recent publication that reported an HPP-like morphologic phenotype, but no expansion of the basal crypt stem cell population upon KRAS mutation in affected crypts [31].

Abi1 was strongly expressed in the mucosal cytoplasm of sessile serrated polyps and adenomas (SSP/A), traditional serrated adenomas (TSA) and tubular adenomas (TbA) compared to healthy mucosa, wild-type and BRAF-mutated HPP. Colorectal carcinoma and colorectal carcinoma metastases showed the strongest cytoplasmic Abi1 staining with no difference between microsatellite-stable (MSS) and -instable (MSI) tumors but with significant overexpression of Abi1 in KRAS-mutated carcinomas compared to healthy and inflamed mucosa, wild-type and BRAF-mutated HPP and SSP/A as well as wild-type tubular adenomas and wild-type invasive carcinomas. These findings are contradictory to results that were recently published by Baba et al, whose group found downregulation of Abi1 isoforms in different intestinal cancers compared to mucosa [32]. However, their results show a different extent of downregulation among the Abi1 isoforms and among different cancers, with the results from colon cancer being not as clear-cut as the results from stomach cancer. Furthermore, KRAS/BRAF mutation and inflammation status as well as histologic grade had not been evaluated in that study.

Our results point rather toward a cytoplasmic upregulation of Abi1 during colorectal tumorigenesis but interestingly, overexpression in KRAS-mutated carcinoma and metastasis was not significant compared to KRAS-mutated precursor lesions. So, in all precursor lesions and invasive carcinomas, activating KRAS mutations led to a significant Abi1 overexpression against the background of the already existing upregulation in consequence of adenomatous change, perhaps related to enhanced PI3K/Akt signalling in these lesions [33]. However, it is still questionable why there was no significant difference in Abi1 expression between wild-type and KRAS-mutated adenomas (SSA/P, TSA and TbA). In our opinion, since the DNA for mutation testing was extracted from a whole biopsy specimen, this finding might be due to the fact that the fraction of KRAS-mutated cells in these precursor lesions might be lower than in a specimen that consists mainly of homogeneous tumor tissue, and Abi1 upregulation upon adenomatous change in major parts of the polyp might mask the stronger Abi1-overexpression in a minor, KRAS-mutated part of the lesion. This would also explain the strong upregulation that could be detected among hyperplastic polyps upon KRAS mutation, since these non-adenomatous lesions lack a “background” of Abi1 expression.

### In Vitro Experiments

In colorectal cancer cell lines, there was strong expression of Abi1 in lysates from KRAS-mutated cells, but only a very faint signal in lysate from BRAF-mutated cells. The appearance of a double-banded signal at 65 kD in Abi1 immunoblotting has been previously described and most likely represents different phosphorylation states of the protein [12], [34]. To further confirm these results, we used two colorectal carcinoma cell lines (CHD-1 and HDC-9) with yet unknown mutation status that have been established by our work group in the 1990s [18], [19] and found that the cell line overexpressing Abi1 (CHD-1) carries an activating mutation in codon 13 of KRAS. Consistent with these findings was the overexpression of Abi1 upon transfection of HDC-9 with mutant KRAS (G12D), while there was no effect on Abi1 expression after transfection with wild-type KRAS. Endogenous Abi1 expression in both cell lines as well as overexpression upon KRAS G12D-transfection in HDC-9 could be suppressed by application of the PI3K-inhibitor Wortmannin. Taken together, our *in vitro* results point towards an upregulation of Abi1 on protein level upon constitutive activation of KRAS that is Wortmannin-sensitive. This is supported by multiple previous studies that characterized the activating effect of oncogenic KRAS on actin dynamics, sometimes through overexpression of proteins that are important for actin reorganization [35]. For example, it has been described that KRAS induces the activity of the small GTPase Rac, thus modulating colorectal cancer cell adhesion and motility [36], [37]. Rac, on the other hand, has been characterized as a positive regulator of actin assembly driven by WAVE family proteins and the associated Abi1/Eps8/Sos1 complex [9], [38].

### Abi1 Overexpression in Inflammation and Cancer

**Figure 4:**
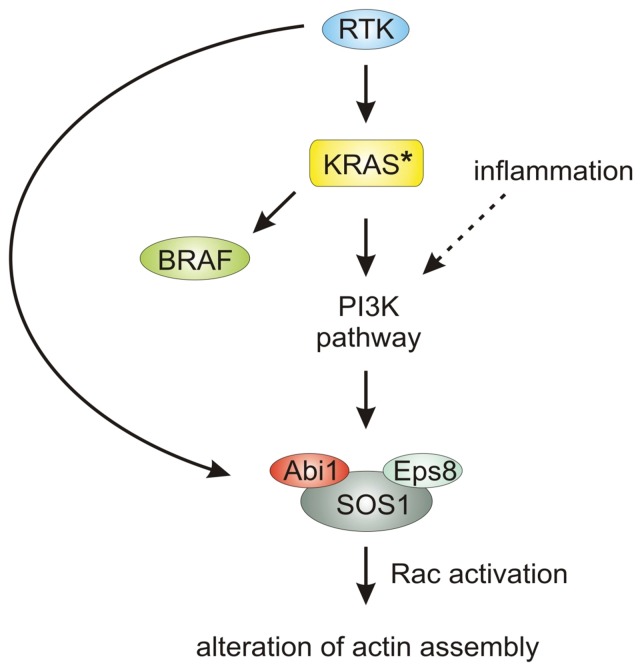
Proposed model for the regulation of actin dynamics via KRAS, PI3K and Abi1. Ligand-binding to membrane-associated receptor tyrosine kinase (RTK) leads to an activation of RAS, which is constitutively activated in mutant KRAS (*). Activated KRAS activates - among others - both the B1 Rapidly accelerated fibrosarcoma (BRAF)- and Phosphatidylinositol-3-kinase (PI3K)-pathway, only the latter leading to activation of the Abi1/Sos1/Eps8 complex. Via activation of the small GTPase Rac, this leads to reorganization of the actin cytoskeleton and to a change in cellular shape. *RTK: receptor tyrosine kinase; KRAS: Kirsten rat sarcoma; BRAF: B1 Rapidly accelerated fibrosarcoma*; *PI3K: Phosphatidylinositol-3-kinase; Abi1: Abelson interactor 1; Eps8: Epidermal growth factor receptor kinase substrate*; *Sos1: Son of sevenless homolog 1*; *Rac: Ras-related C3 botulinum toxin substrate.*

KRAS-induced upregulation of Abi1 via the Phosphatidylinositol-3-kinase (PI3K)-pathway might explain the BRAF- independence of Abi1 expression shown in patient samples and cell lines and the suppressive effect of Wortmannin. A schematic model for this possible regulatory pathway is depicted in Fig. 4. Noteworthy, PI3K has already been shown to form a complex with Abi1, thus enhancing Rac activity [39], [40], [10]. This hypothesis would also explain the observed upregulation of Abi1 upon inflammation in colonic mucosa since PI3K is a key player in inflammatory response [41]. Consistent with that, we could show increased Akt phosphorylation and overexpression of Abi1 upon TNFalpha stimulation in KRAS wild-type colorectal carcinoma cells. Given that, a BRAF-independent upregulation of Abi1 via the PI3K-pathway as a target protein in actin dynamics downstream from KRAS is easily conceivable. Our finding of Abi1 overexpression during the development of invasive carcinomas is comparable to findings made in ovarian cancer, where coexpression of the trimeric Abi1/SOS1/Eps8-complex is a prerequisite for Rac-dependent ovarial cancer cell motility upon lysophosphatidic acid (LPA)-stimulation [42]. Furthermore, Abi1 has been shown to be a positive regulator of breast cancer cell proliferation, migration and invasion [15]. For metastases, Iwaya et al. showed in 2007 that overexpression of the Abi1/WAVE-complex-interactor Arp2 as well as protein interaction of Arp2/3 with WAVE2 in colonic carcinoma cells promotes metastasis and introduction of an activating KRAS mutation enhances the ability of colon adenocarcinoma cells to migrate and invade through filopodia formation and PI3K-dependent Cdc42 activation [43], [44] Therefore, Abi1 expression might be associated with epithelial-mesenchymal transition (EMT) of colon cancer cells as a prerequisite to invasion and metastasis and consistent with that, the observed downregulation of Abi1 in established metastases might be due to a reversal of that process in the context of mesenchymal-epithelial transition (MET) at the site of metastasis [45]. Compatible with our findings of low Abi1-expression in BRAF-mutated lesions, the MET process has just recently been linked to increased RAF signalling in metastatic breast carcinoma [46].

Noteworthy Abi1 was detected cytoplasmically, but not in the nucleus by immunohistochemistry. However, in CHD-1 colorectal carcinoma cells carrying the KRAS G13D mutation, we could show slight nuclear Abi1 positivity in immunofluorescence microscopy. This is interesting because it has previously been shown that Abi1 is able to shuttle into the nucleus in a phosphorylation-dependent manner and to interact with nuclear transcription factors of the Myc/Max family [47], [13]. Results obtained in this study, however, point rather towards a cytoplasmic role for Abi1, perhaps as part of the previously mentioned trimeric Abi1/SOS1/Eps8-complex downstream of PI3K signalling. Since most of the KRAS-mutated lesions analyzed by immunohistochemistry in this study carried the more frequent codon 12 mutation, it could be argued that there might be different signalling patterns in carcinoma cells carrying activating mutations in codon 13 that might lead to nuclear translocation of Abi1. This would be supported by recent studies which describe a different biological behaviour of carcinomas with KRAS codon 13 compared to other mutations [48], [49].

Taken together, it has to be pointed out that the upregulation of Abi1 that we observed is not restricted to carcinogenesis, but can also be observed in inflammation and in very early lesions that do not necessarily progress to cancer, such as hyperplastic polyps. In these polyps and in colorectal carcinomas, there is significant overexpression of the protein in KRAS-mutated lesions compared to both wild-type and BRAF-mutated controls. Our findings might be explained by a common mechanism of Abi1 upregulation via the PI3K-pathway for which we provide first *in vitro* evidence. Furthermore, our findings raise the question of a possible role for Abi1 in actin dynamics downstream of PI3K during inflammation and colonic carcinogenesis and finally, indicate a possible usefulness for Abi1 immunohistochemistry as a marker for early KRAS mutation in otherwise inconspicuous hyperplastic polyps.

## Materials and Methods

### Objectives

The objective of this study was

- to analyze Abi1 expression in 33 mucosal samples, 23 hyperplastic polyps, 20 sessile serrated polyps/adenomas, 8 traditional serrated adenomas and 13 tubular adenomas as well as samples from 20 colorectal carcinomas and 9 colorectal carcinoma metastases by immunohistochemistry;
- to analyze KRAS codon 12/13 and BRAF codon 600 mutation status as well as expression of mismatch repair proteins MLH1, PMS2, MLH2 and MSH6 of all included precursor lesions, carcinomas and metastases;
- to evaluate differences in Abi1 expression by statistical hypothesis testing;
- to analyze Abi1 expression in 3 established colorectal carcinoma cell lines with known KRAS/BRAF mutation and microsatellite status by western immunoblotting
- to analyze Abi1 expression and KRAS/BRAF mutation status in two additional colorectal carcinoma cell lines (CHD-1 and HDC-9) that had previously been established by our research group by western immunoblotting, KRAS/BRAF strip assay testing and DNA pyrosequencing;
- to investigate the effect of KRAS wild-type and KRAS G12D transfection as well as treatment with TNFalpha on the expression of Abi1 and on the phosphorylation status of Akt and Erk1/2 in KRAS wild-type HDC-9 colorectal carcinoma cells
- to investigate the effect of the PI3K-inhibitor Wortmannin on endogenous Abi1 expression in CHD-1 and HDC-9 as well as after transfection of KRAS wild-type HDC-9 cells with mutant KRAS.

### Tissue Samples

There were 95 patients and 126 tissue samples in the study. All samples were submitted to the Institute of Pathology of the University of Ulm, to the Dept. of Pathology of the Bundeswehrkrankenhaus Ulm, to the Gemeinschaftspraxis for Pathology, Augsburg or to the Institute of Pathology, Klinikum Augsburg, for diagnostic purpose from January 1^st^, 2006 to December, 31^st^, 2009. Healthy mucosa samples were obtained from routine colonoscopies, while inflamed mucosa samples were obtained from patients with acute episodes of inflammatory bowel disease (Morbus Crohn or Ulcerative colitis) without evidence for dysplasia. The specimens were fixed in 10% buffered formalin and the whole specimen or representative blocks embedded in wax and sections stained with haematoxylin and eosin. For this study, hyperplastic polyps (HPP), sessile serrated polyps/adenomas (SSA/P), traditional serrated adenomas (TSA) and tubular adenomas (TbA) have been re-reviewed and assigned to a diagnosis group according to the morphologic criteria reviewed by Snover in 2011 [4] (K.S., M.J.S, B.M. and K.K.). We refrained from further sub-dividing hyperplastic polyps morphologically. Sessile serrated polyps/adenomas with conventional dysplasia or mucosal carcinoma/carcinoma in situ were excluded from the study. The same criteria were established for traditional serrated adenomas and tubular adenomas. Carcinoma samples were taken from surgical resection specimens and metastasis samples were taken from diagnostic biopsies. Nuclear expression of mismatch repair proteins MLH1, PMS2, MLH2, and MSH6 was shown by immunohistochemistry in precursor lesions, carcinomas and metastases, indicating microsatellite stability (MSS) in all examined precursor lesions, metastases and in 17 of 20 invasive colorectal carcinomas. 3 carcinomas showed loss of MLH1 and PMS2 and were therefore regarded as microsatellite instable (MSI). The clinico-pathological characteristics (age, gender, localization of sampling, sample size, mutation status, and – if applicable - tumor grade, tumor stage and localization of metastases) of specimens are summarized in Table 1.

### Immunohistochemistry, Image Acquisition and Expression Analysis

Immunohistochemistry was done as previously described [17]. Mouse monoclonal antibody against Abi1 was obtained from MBL (Woburn, USA), diluted in antibody diluent (conc. 1∶200; Zytomed Systems, Berlin, Germany) and applied for 60 min at room temperature. After application of washing buffer (Zytomed), peroxidase-blocking for 5 min (Zytomed) and a single 2 min buffer wash, AP-Polymer (Zytomed) was applied for 30 min at room temperature and followed by further washing with buffer to remove unbound antibody. Sites of antibody binding were then detected by Permanent AP Red Chromogen (Zytomed). Other immunostainings were performed on a BenchMark Autostainer (Ventana Medical Systems, Tucson, USA). Ready-to-use rabbit monoclonal primary antibodies (Ki-67 (clone 30–9), CDX-2 (clone EPR2764Y), Cytokeratin 20 (clone SP33) and Cytokeratin 7 (clone SP52) optimally diluted according to the manufacturer’s recommendations were purchased from Ventana Medical Systems (Tucson, USA). Finally, sections were washed in water, lightly counterstained with haematoxylin, dehydrated and mounted. Omitting the primary antibody from the immunohistochemical procedure and replacing it by antibody diluent acted as negative control. The sections were evaluated by light microscopic examination using a Leica DM6000B light microscope (Leica, Wetzlar, Germany). The intensity of immunostaining in each section was assessed independently by two observers (K.S. and B.M., blinded to mutation status) using the scoring system described. Both the intensity of immunostaining and the proportion of positively stained cells were assessed. The intensity of immunostaining was graded as negative = 0, weak = 1, moderate = 2 or strong = 3 [50]. The proportion of positively stained cells was assessed as no cells = 0, 1–25% of cells = 1, 26–50% = 2, 51–75% = 3 and 76–100% = 4. The numbers representing intensity and percentage of stained cells were added together and the result will be referred to as score. Image acquisition was performed using a Leica DM6000B light microscope (Leica, Wetzlar, Germany) and the Diskus Mikroskopische Diskussion image acquisition software (Carl H. Hilgers, Königswinter, Germany).

### Statistics

Expression level differences between diagnostic groups were assessed by t-test for unpaired data. Testing was performed with Microsoft Excel (Microsoft, Seattle, USA). All *p-*values were considered two-tailed and 0.1 was used as upper threshold for statistical significance. Graphical display in box and whisker plot was done using the Box Plot Template for Excel by Vertex42 (Albuquerque, USA – www.vertex42.com).

### KRAS and BRAF Mutation Analysis

We examined all precursor lesions (HPP, SSA/P, TSA, TbA), carcinomas and metastases as well as the 5 included colorectal carcinoma cell lines for mutation status of KRAS and BRAF. Samples were excluded when quantity or quality of the material did not allow DNA extraction or mutation analysis, or the results of mutation analysis were not clear-cut. DNA was extracted from 30 µm-thick slides that were cut off paraffin blocks, xylene treated and ethanol washed as previously described [51]. PCR amplification of KRAS and BRAF with biotinylated primers and hybridization-based mutation analysis was performed using the KRAS/BRAF strip assay (Vienna Labs, Vienna, Austria and AID diagnostics, Strassberg, Germany) according to the manufacturers’ protocols. For comparison, we grouped the activating mutations as follows: KRAS codon 12 (Gly>Ala, Arg, Asp, Cys, Ile, Leu, Ser, Val) and KRAS codon 13 (Gly>Asp,Cys) as KRASc12/13 and BRAF codon 600 (Val>Glu) as BRAFc600. KRAS/BRAF mutation analysis in cell lines was repeated by DNA pyrosequencing. Therefore, cells were lysed in standard lysis buffer and single-stranded DNA was created using the PyroMark Q24 Vacuum Workstation (Qiagen, Hilden, Germany). Pyrosequencing was then performed on the PyroMark Q24 System using the therascreen KRAS and BRAF Pyro Kits (all Qiagen, Hilden, Germany) according to the manfacturer’s protocol. Data analysis and creation of pyrograms was done using the PyroMark Q24 Software 2.0 (Qiagen, Hilden, Germany).

### Cell Culture, Transfection Experiments and Western Blot

Human colorectal carcinoma cell lines SW620, SW1116 and Colo205 were obtained from ATCC (Rockville, USA). Colon carcinoma cell lines CHD-1 and HDC-9 were established from freshly isolated tumor cells by one of us (S.B.) and have been previously characterized [18], [19]. Derivation of CHD-1 and HDC-9 from colorectal carcinoma was reassured by immunohistochemistry for CDX2, Cytokeratin 7 and Cytokeratin 20 (Fig. S2). SW620, SW1116 and Colo205 are microsatellite stable (MSS), for CHD-1 and HDC-9 microsatellite stability was proven by positive nuclear immunostaining of mismatch repair proteins MLH1, PMS2, MLH2, and MSH6 (data not shown). Cells were maintained in DMEM supplemented with 10% FCS. Transfection experiments were performed using the Optifect transfection reagent (Invitrogen, Karlsruhe, Germany) according to the manufacturer’s recommendations 36 hours prior to cell lysis in medium without serum and antibiotics. TNFalpha (SignalChem, Richmond, Canada) was added in 2 doses of each 10 ng 24 and 12 hours prior to cell lysis. Wortmannin (AppliChem, Darmstadt, Germany) was added to a final concentration of 50 nM for 72 hours after KRAS transfection. Cells cultured at normal conditions and/or after transfection/TNFalpha/Wortmannin treatment were lysed in standard lysis buffer containing kinase and phosphatase inhibitors. The supernatants were collected by centrifugation at 10.000 x g for 5 min at 4°C. Overexpression of KRAS was tested by Ras immunoblotting, and successful insertion of mutated KRAS into HDC-9 cells was proven by mutation testing using the KRAS/BRAF strip assay and DNA pyrosequencing (Fig. 3C, right lane and Fig. S3, lower left pyrogram). For protein detection, 10 µg of total protein was separated by a 10% SDS gel. Immunoblot analysis was done according to standard methods using the following antibodies: anti-Abi1 (mouse monoclonal, conc. 1∶1.000, MBL, Woburn, USA), anti-PI3K(p85) (mouse monoclonal, conc. 1∶1.000, Abcam, Cambridge, UK), anti-Ras (rabbit monoclonal, conc. 1∶1000, Cell Signaling Tech., Boston, USA), anti-phospho-Akt(Ser473) (rabbit monoclonal, conc. 1∶1000, Cell Signaling Tech., Boston, USA) and anti-phospho-p44/42 MAPK (Erk1/2) (rabbit monoclonal, conc. 1∶1000, Cell Signaling Tech., Boston, USA). ß-Actin was used as a loading control (conc. 1∶10.000, mouse monoclonal antibody against ß-Actin, Abcam, Cambridge, UK).

### Expression Vectors

The pcDNA3-KRAS-wild type and pcDNA3-KRAS-G12D vectors were a kind gift of Dr. Patrizio Castagnola, National Cancer Research Center, Genova, Italy, and have been previously published [52]. The constructs contain either the complete cds sequences for KRAS wild-type or KRASG12D (GGT/GAT transition) inserted in a mammalian pcDNA3.1 expression vector.

### Immunofluorescence Microscopy

Immunocytochemistry was performed as previously described by our work group [13]. In brief, cultured cells were fixed with ice-cold Methanol and permeabilized in a buffer containing 0.2% Triton-X-100/0.1% Na-Citrate/PBS. Blocking was then performed with 10% FCS/PBS for 1 h at RT followed by one hour incubation with the primary and secondary antibodies and mounting in vectashield aqueous mount (Vector, USA). Cell nuclei were counterstained with 4,6-diamidino-2-phenylindole (DAPI). The primary antibody was anti-Abi-1 (mouse monoclonal, conc. 1∶250, MBL, Woburn, USA). The fluorescence-labeled secondary antibody was Alexa FluorH 568 (red, used filter set: excitation BP 534 nm–558 nm, FT 560, emission BP 575 - 640). Image acquisition was performed using a Leica DM6000B light microscope (Leica, Wetzlar, Germany) and the Diskus Mikroskopische Diskussion image acquisition software (Carl H. Hilgers, Königswinter, Germany).

### Ethics

All tissue samples were collected for histologic examination and diagnosis purpose and anonymized for the use in this study. Informed consent was therefore not needed to be obtained. This was approved by the ethics committee of the University of Ulm and conforms to the current guidelines of the German Ethics Council [53].

## Supporting Information

Figure S1 **Ki67 expression in KRAS-wild type and KRAS-mutated hyperplastic polyps. A** and **B**, both polyps show only basal positivity for Ki67. There is no expansion of the proliferative zone in KRAS-mutated HPP. *Stain: anti-Ki67, haematoxylin; Bar indicates 200 µm*.
(TIF)

Figure S2 **Immunohistochemical characterization of CHD-1 and HDC-9 cells.** Both cell lines stain positive for CDX-2 and cytokeratin 20 and negative for cytokeratin 7. *Stain: haematoxylin/eosin, anti-CDX2, anti-cytokeratin 7, anti-cytokeratin 20 as indicated; Bar indicates 25 µm*.
(TIF)

Figure S3 **Pyrosequencing data from CHD-1 and HDC-9 cells.** The upper left pyrogram shows an activating G13D mutation in the CHD-1 cell line (arrowhead), while HDC-9 cells are KRAS wild-type (central left pyrogram). Transfection of HDC-9 cells with a KRAS G12D-construct leads to appearance of a small peak, indicating a KRAS G12D-mutation (lower left pyrogram, arrowhead). Both cell lines are BRAF wild-type (right pyrograms).
(TIF)

Figure S4 **Exact p-values from statistical hypothesis testing.** See Fig. 3 B for graphical display.
(TIF)

Table S1 **KRAS and BRAF mutations in analyzed samples.**
(TIF)
