# Supplementary figures and images for: Expression of Abelson Interactor 1 (Abi1) Correlates with Inflammation, KRAS Mutation and Adenomatous Change during Colonic Carcinogenesis
Source: PLoS One. 2012 Jul 10;7(7):e40671. doi: 10.1371/journal.pone.0040671 (PMC3393686; doi:10.1371/journal.pone.0040671)

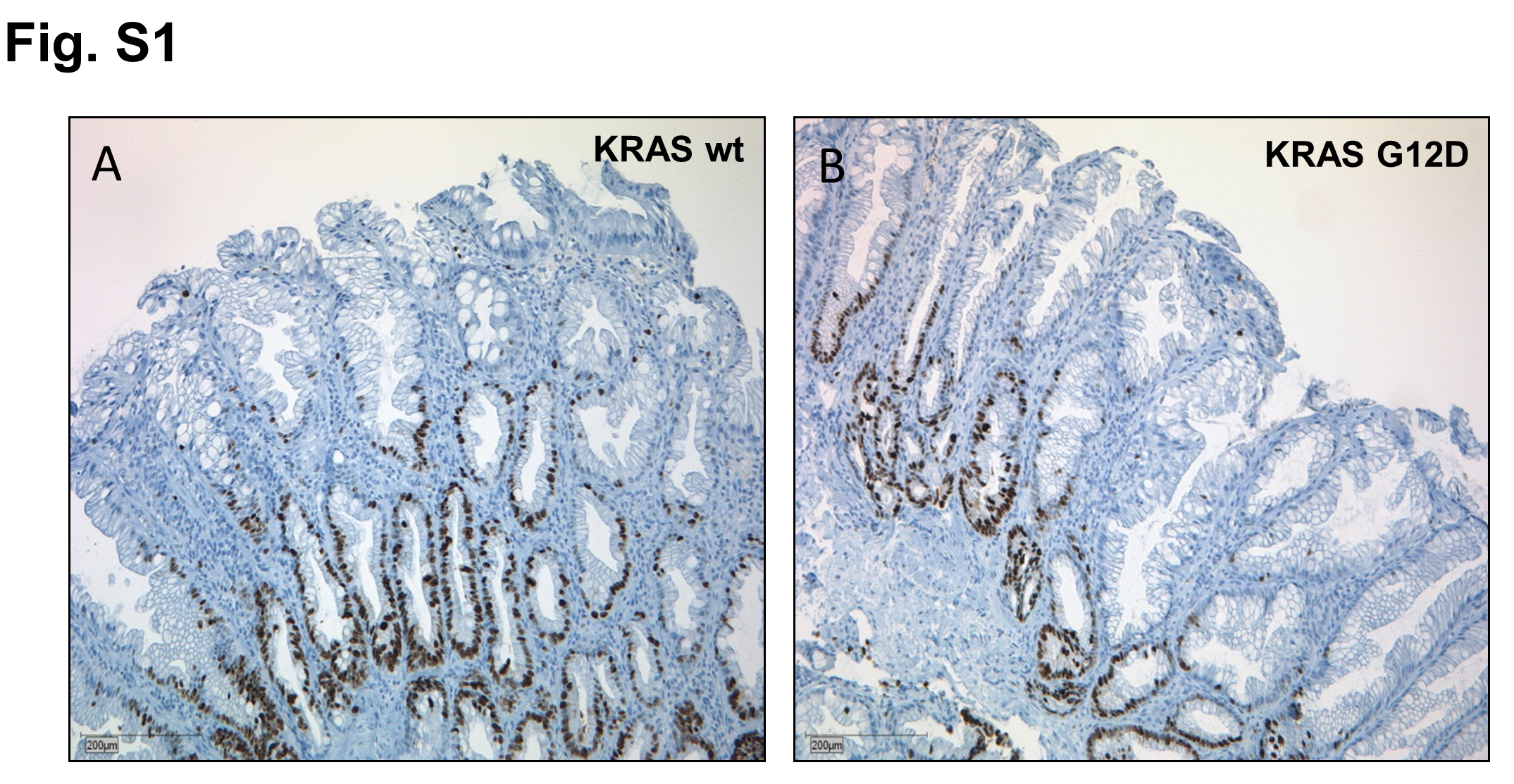

Supplement: Figure S1 — Ki67 expression in KRAS-wild type and KRAS-mutated hyperplastic polyps. A and B, both polyps show only basal positivity for Ki67. There is no expansion of the proliferative zone in KRAS-mutated HPP. Stain: anti-Ki67, haematoxylin; Bar indicates 200 µm. (TIF) [file pone.0040671.s001.tif]

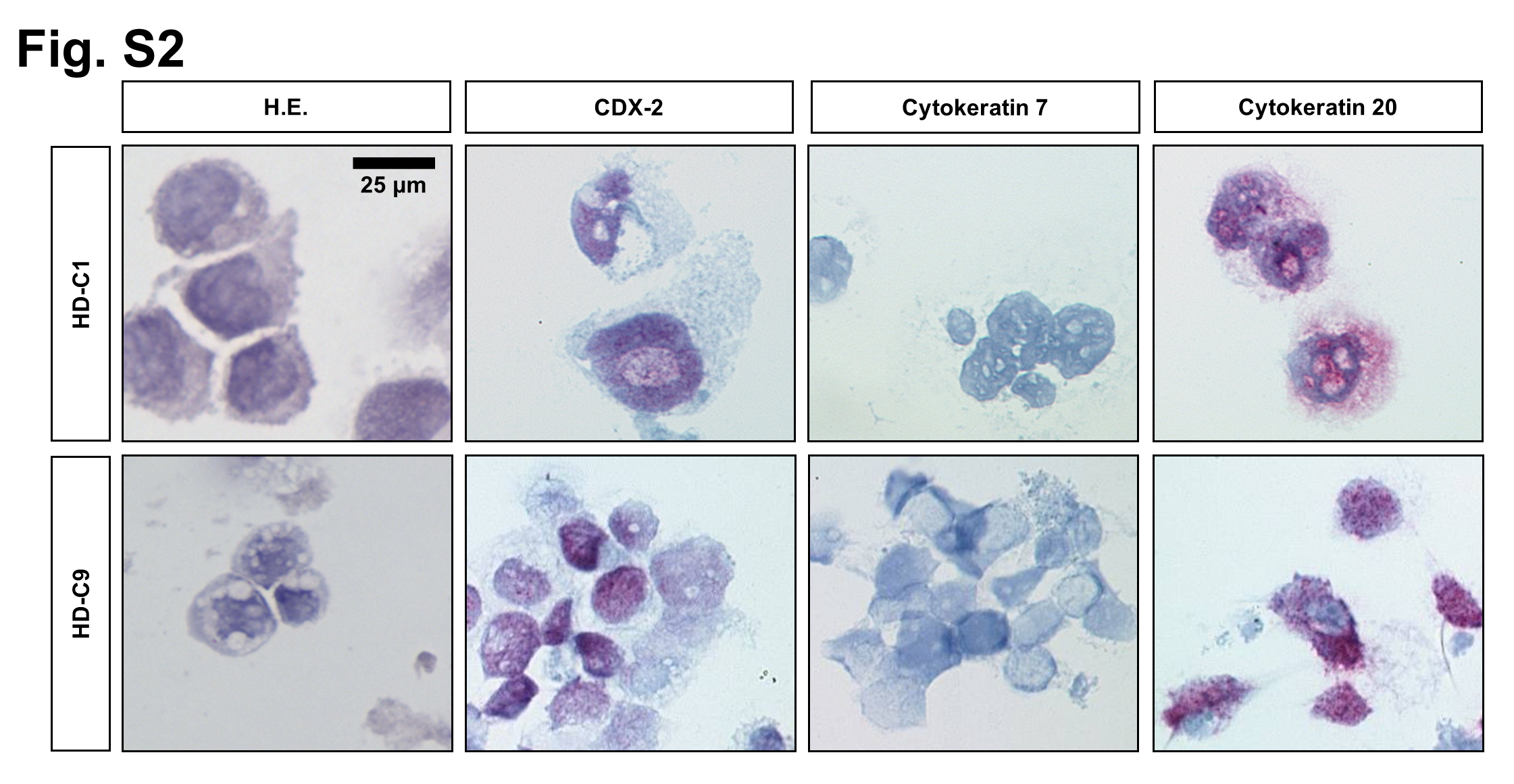

Supplement: Figure S2 — Immunohistochemical characterization of CHD-1 and HDC-9 cells. Both cell lines stain positive for CDX-2 and cytokeratin 20 and negative for cytokeratin 7. Stain: haematoxylin/eosin, anti-CDX2, anti-cytokeratin 7, anti-cytokeratin 20 as indicated; Bar indicates 25 µm. (TIF) [file pone.0040671.s002.tif]

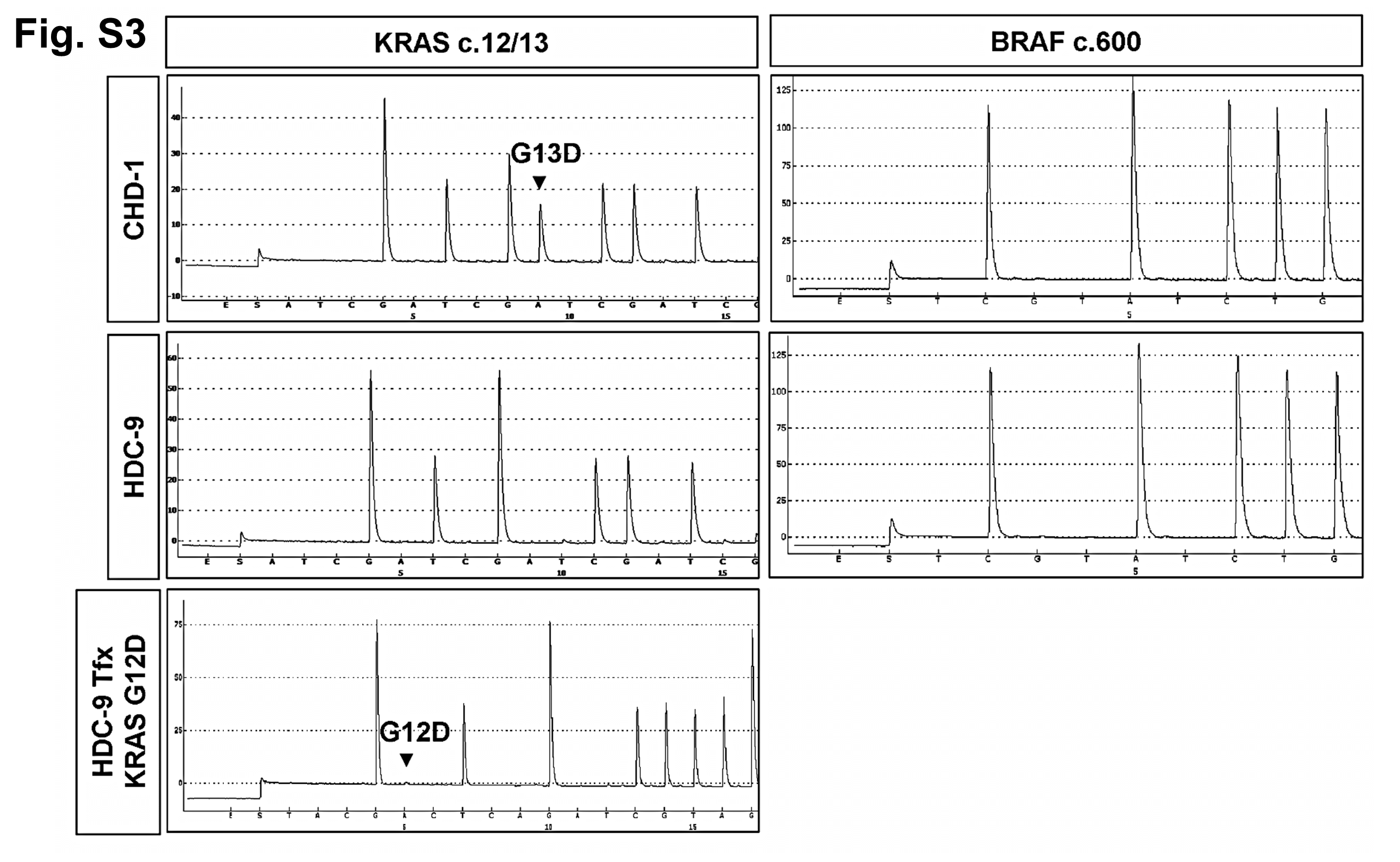

Supplement: Figure S3 — Pyrosequencing data from CHD-1 and HDC-9 cells. The upper left pyrogram shows an activating G13D mutation in the CHD-1 cell line (arrowhead), while HDC-9 cells are KRAS wild-type (central left pyrogram). Transfection of HDC-9 cells with a KRAS G12D-construct leads to appearance of a small peak, indicating a KRAS G12D-mutation (lower left pyrogram, arrowhead). Both cell lines are BRAF wild-type (right pyrograms). (TIF) [file pone.0040671.s003.tif]
